# Covid-19 Hospitalization Costs Assessment in an Italian Covid Hospital: Marco Scattaglia

**DOI:** 10.1093/eurpub/ckac131.071

**Published:** 2022-10-25

**Authors:** M Scattaglia, GA Campobasso, A Cremona, M Morandi, MM Gianino

**Affiliations:** 1 Department of Sciences of Public Health and Pediatrics, University of Turin, Turin, Italy; 2 Martini Hospital, ASL Città di Torino, Turin, Italy; 3 San Giovanni Bosco Hospital, ASL Città di Torino, Turin, Italy

## Abstract

**Introduction:**

Many consequences resulted from the breakout of the COVID-19 pandemic in Europe, which have disrupted our economic, social and medical world. This allowed us to measure and assess the hospitalisation costs regarding the COVID-19 disease at Martini Hospital in Turin, one of the hospitals entirely committed to the COVID-19 care, between January and June 2021.

**Methods:**

In this single center retrospective study, we collected and analysed cost data on patients admitted at Martini Hospital in the time frame of January-June 2021 and compared the analysis with the same period in 2020, at a time when the hospital was not dedicated to Covid-19 patients. Cost data included full-time and temporary employees salaries, drugs, medical and non-medical supplies and equipment and facility utilities. We then estimated the cost per treated COVID-19 episode, in comparison with the cost per any desease including Covid-19.

**Results:**

The first 6 months of 2021 registered 2,136 hospital discharges, while same period in 2020 counted 4376. The mean duration of the hospital stay was 7,67 days in 2020 and 12,83 in 2021. The average charge per treated episode doubled (+52,5%) from Euros 8997 in 2020 to Euros 19026 in 2021. The mean revenue increased of 35% from Euros 3280 in 2020 to Euros 5041 in 2021. This is due to the major complexity of care required for Covid patients. As it is, in 2021 the average complexity index of 2.13 while in 2020 it was 1.39.

**Conclusions:**

Clinical management and treatment of COVID-19 economically strain the European health-care systems. The study of COVID-19 treatment costs, and their differences between 2020 and 2021 suggests an economic challenge for the entire Italian health system and emphasises the necessity to avoid the recurrence of such an economic impact by implementing effective infection prevention and control policies.

**Key messages:**

• The Covid-19 pandemic has been straining both the European health and economic systems.

• Studying the Covid-19 expenditures allows to frame unexpected new challenges regarding health-care systems.

